# THP-1 cells transduced with CD16A utilize Fcγ receptor I and III in the phagocytosis of IgG-sensitized human erythrocytes and platelets

**DOI:** 10.1371/journal.pone.0278365

**Published:** 2022-12-14

**Authors:** Lazaro Gil Gonzalez, Yuniel Fernandez-Marrero, Peter Alan Albert Norris, Zoya Tawhidi, Yuexin Shan, Yoelys Cruz-Leal, Kevin Doyoon Won, Kayluz Frias-Boligan, Donald R. Branch, Alan H. Lazarus

**Affiliations:** 1 Keenan Research Centre for Biomedical Science, St. Michael’s Hospital, Unity Health Toronto, Toronto ON, Canada; 2 Biological Sciences, Sunnybrook Research Institute, Toronto, ON, Canada; 3 Innovation and Portfolio Management, Canadian Blood Services, Ottawa, ON, Canada; 4 Department of Laboratory Medicine and Pathobiology, University of Toronto, Toronto, ON, Canada; 5 Department of Medicine, University of Toronto, Toronto, ON, Canada; Sungkyunkwan University and Samsung Advanced Institute of Health Science and Technology (SAIHST), REPUBLIC OF KOREA

## Abstract

Fc gamma receptors (FcγRs) are critical effector receptors for immunoglobulin G (IgG) antibodies. On macrophages, FcγRs mediate multiple effector functions, including phagocytosis, but the individual contribution of specific FcγRs to phagocytosis has not been fully characterized. Primary human macrophage populations, such as splenic macrophages, can express FcγRI, FcγRIIA, and FcγRIIIA. However, there is currently no widely available monocyte or macrophage cell line expressing all these receptors. Common sources of monocytes for differentiation into macrophages, such as human peripheral blood monocytes and the monocytic leukemia cell line THP-1, generally lack the expression of FcγRIIIA (CD16A). Here, we utilized a lentiviral system to generate THP-1 cells stably expressing human FcγRIIIA (CD16F158). THP-1-CD16A cells treated with phorbol 12-myristate 13-acetate for 24 hours phagocytosed anti-D-opsonized human red blood cells primarily utilizing FcγRI with a lesser but significant contribution of IIIA while phagocytosis of antibody-opsonized human platelets equally utilized FcγRI and Fcγ IIIA. Despite the well-known ability of FcγRIIA to bind IgG in cell free systems, this receptor did not appear to be involved in either RBC or platelet phagocytosis. These transgenic cells may constitute a valuable tool for studying macrophage FcγR utilization and function.

## Introduction

Fc gamma receptors (FcγRs) are a family of receptors expressed on the surface of many immune cells that recognize and engage the Fc portion of immunoglobulin G (IgG) [1,2]. FcγRs can be broadly categorized as activating or inhibitory as defined by the presence of intracellular signaling motifs. In humans, classical activating FcγRs include FcγRI/CD64, FcγRIIA/CD32A, FcγRIIC/CD32C, and FcγRIIIA/CD16A. Activating FcγRs signal through an immunoreceptor tyrosine-based activation motif (ITAM), present in their intracytoplasmic domain (FcγRIIA and FcγRIIC) or via an associated Fc receptor gamma (FcRγ) chain (FcγRI and FcγRIIIA) [1]. ITAM signaling drives the activation of signaling cascades that can promote pleiotropic cell effects including activation, degranulation, proliferation, endocytosis, or phagocytosis [3,4]. In contrast, FcγRIIB/CD32B bears an immunoreceptor tyrosine-based inhibition motif (ITIM) in its intracytoplasmic domain. ITIM signaling has been considered inhibitory by its ability to recruit phosphatases that antagonize the signaling mediated by activating FcγRs [5]. FcγRs play beneficial roles in controlling infections and mediating anti-tumor responses, but also pathogenic roles in autoimmune and inflammatory diseases [6,7].

Macrophages are mononuclear phagocytes that are widely distributed throughout the body as tissue-specific subpopulations, contributing to homeostasis, and participating in innate and adaptive immune responses [8,9]. Across subpopulations, macrophages can express all FcγRs except for FcγRIIIB, which is expressed on neutrophils and on some subsets of basophils [10,11]. Macrophage FcγRs can mediate the phagocytosis of antibody-opsonized pathogens as well as the killing of virally infected cells or tumor cells through antibody-dependent cellular cytotoxicity (ADCC) [12]. However, the engagement of FcγRs on macrophages can also lead to the destruction of autoantibody-opsonized cells in autoimmunity, such as erythrocytes in autoimmune hemolytic anemia [13–15] or platelets in immune thrombocytopenia (ITP) [16–18]. In addition to autoimmune RBC and platelet destruction, these cells can also be destroyed by alloantibodies produced as a result of transfusion or pregnancy [19,20].

FcγRI is a receptor with a high relative affinity for IgG and can mediate macrophage phagocytosis and internalization of IgG-immune complexes [21]. FcγRIIA has been shown to be critical for mediating macrophage-derived inflammatory cytokine release by antibody-dependent inflammation, but some reports have also shown its involvement in phagocytosis [22,23]. FcγRIIIA on macrophages is critical for killing tumor cells through antibody-dependent cellular cytotoxicity [12]. Both FcγRIIIA and FcγRIIA can also facilitate antibody-dependent enhancement of some viral infections such as dengue [24,25], influenza, ebola, and human immunodeficiency virus (HIV) [26].

Currently, *in vitro* cultures of primary macrophages are used to study FcγRIIIA effector function. We are not aware of a human cell line that expresses this receptor. Unfortunately, common sources of monocytes for macrophage generation, such as THP-1 cells or human peripheral blood monocytes, display limited FcγRIIIA expression [27–29]. In addition, although it has been suggested that FcγRIIIA expression can be induced on THP-1 cells using IFN-γ plus lipopolysaccharide (LPS) [30], we were unable to accomplish this (unpublished observations). Primary cells are also subject to limitations, including the difficulty in isolating a sufficient number of cells for experimentation and the variability associated with donors [31]. The study of FcγRIIIA is therefore somewhat hindered by the lack of a readily available monocyte/macrophage cell line expressing this receptor.

Using a lentiviral system, we transduced THP-1 cells with the genes that encode for the human CD16A molecule (CD16F158) and the FcRγ chain to help promote FcγRIIIA expression. Transduced cells (THP-1-CD16A) showed stable expression of FcγRIIIA in the presence of puromycin without interfering with endogenous FcγR expression. THP-1-CD16A cells that had been differentiated into macrophages phagocytosed anti-D-opsonized human red blood cells to a similar magnitude as wild type THP-1, through the use of both FcγRIIIA and FcγRI. Additionally, these two receptors were equally utilized by THP-1-CD16A cells for phagocytosing antibody-opsonized human platelets. This new THP-1-CD16A cell line (ATCC Accession Number CRL3575) could therefore be a valuable tool for *in vitro* studies of the physiology of FcγRIIIA.

## Materials and methods

### Bacterial strains, cell culture, plasmids, antibodies, and mice

*Escherichia coli* strain DH5Alpha (*F- Φ80lacZΔM15 Δ(lacZYA-argF) U169 recA1 endA1 hsdR17(rk-*, *mk+) phoA supE44 thi-1 gyrA96 relA1 λ-*) purchased from Thermo Fisher Scientific (Ontario, Canada), and *E*. *coli* strain NEB Stable (*F’ proA+B+ lacIq Δ(lacZ)M15 zzf*::*Tn10 (TetR)/ Δ(ara-leu) 7697 araD139 fhuA ΔlacX74 galK16 galE15 e14- Φ80dlacZΔM15 recA1 relA1 endA1 nupG rpsL (StrR) rph spoT1 Δ(mrr-hsdRMS-mcrBC)*) purchased from New England BioLabs (NEB) (Massachusetts, USA), were used for propagating the plasmids. Bacterial strains were grown in LB medium and conserved at -70°C in the same medium supplemented with 20% glycerol. Ampicillin (BioShop, Canada) at a final concentration of 50 μg/ml was added when necessary.

HEK-293T/17 cells (ATCC, CRL-11268) were maintained at 37°C, 5% CO_2_ in DMEM (Gibco, USA) supplemented with 10% fetal bovine serum (FBS) (Wisent Bioproducts, Canada), 100 U/mL penicillin, and 100 μg/mL streptomycin (Gibco, USA). THP-1 cells (ATCC, TIB-202) were maintained at 37°C, 5% CO_2_ in complete RPMI medium consisted of RPMI 1640 (Gibco, USA) supplemented with 10% FBS, 2 mM Gluta-Plus (Wisent Bioproducts, Canada), and 100 U/mL penicillin, 100 μg/mL streptomycin. Cells were cultured in 75 cm^2^ Nunc cell culture treated EasYFlasks (Thermo Fisher Scientific, Denmark).

The plasmids pUC19 (plasmid #500050), a gift from Joachim Messing to Addgene (Massachusetts, USA) was used for the cloning strategy and the plasmid pVLX-puro from Clontech Laboratories (California, USA) was used for the construction of the lentiviral transfer plasmid. Plasmids pMD2.G (plasmid #12259, VSV-G envelope expressing vector) and psPAX2 (plasmid # 12260, 2nd generation lentiviral packaging vector), both a gift from Didier Trono to Addgene (Massachusetts, USA), were also used for transfection of HEK-293T17 cells to produce the lentivirus. Plasmids phFcgRIIIA/Zeocin and phFcεR1/Blasticidin were previously generated in our lab containing the genetic sequence for FcγRIIIA and the Fc receptor gamma chain (FcRγ) respectively.

Purified mouse anti-human CD16 (clone 3G8), purified mouse anti-human CD64 (clone 10.1), BV421-conjugated mouse anti-human CD16 (clone 3G8), BV421-conjugated mouse IgG1 isotype control (clone MOPC-21), PE/Cy7-conjugated mouse anti-human CD64 (clone 10.1), PE/Cy7-conjugated mouse IgG1 isotype control (clone MOPC-21), APC-conjugated mouse anti-human CD14 (clone HCD14) and purified mouse anti-human GPIIb/IIIa (clone A2A9/6) were purchased from BioLegend (California, USA). Purified mouse anti-human CD32 (clone AT10), Alexa Fluor 647-conjugated mouse anti-human CD32 (clone AT10), and Alexa Fluor 647-conjugated mouse IgG1 isotype control (clone 111711) were purchased from Novus Biologicals Canada (Ontario, Canada). Purified mouse anti-human CD32 (clone IV.3), FITC-conjugated mouse anti-human CD32 (clone IV.3), and FITC-conjugated mouse IgG2b isotype control (clone MPC-11) were purchased from Stemcell Technologies Inc. (British Columbia, Canada). Purified mouse IgG1 (clone MOPC-21) and IgG2b (clone MPC-11) isotype controls with an unknown specificity were purchased from BioXCell (New Hampshire, USA). Alexa Fluor 647-conjugated mouse anti-human CD42a (clone GRP-P) was purchased from BioRad Laboratories (California, USA).

### Cloning strategy and lentivirus transduction

The genetic sequences of the FcγRIIIA (CD16F158) and FcRγ (Fc receptor γ chain) were amplified by polymerase chain reaction (PCR) using Phusion high-fidelity DNA polymerase (NEB) and designed primers. The plasmids phFcγRIIIA/Zeocin and phFcεR1/Blasticidin were used as templates. The amplified fragments FcγRIIIA and FcRγ were phosphorylated with T4 polynucleotide kinase (PNK) (NEB), then sequentially cloned in the KpnI and SmaI sites of the pUC19 vector, respectively, to generate the plasmid pUC19-FcγRIIIA-FcRγ. Following a fast-cloning procedure as previously described [32], a genetic sequence that encodes for 2A self-cleaving peptides (P2A) [33] was introduced between the sequences that encode for FcγRIIIA and FcRγ. PCR was performed using the Phusion high-fidelity DNA polymerase (NEB) and the plasmid pUC19-FcγRIIIA-FcRγ was used as the template. The resulting product was ligated with T4 DNA ligase (NEB) to obtain the plasmid pUC19-FcγRIIIA-P2A-FcRγ. The fragment FcγRIIIA-P2A-FcRγ was extracted with restriction enzymes EcoRI-high fidelity (HF) and /XbaI (NEB), then ligated into the EcoRI/XbaI sites of pVLX-puro plasmid to generate the pVLX-puro- FcγRIIIA-P2A-FcRγ.

HEK-293T/17 cells were transfected with the plasmids pMD2-G, psPAX2 and pVLX-puro- FcγRIIIA-P2A-FcRγ using a plasmid ratio of 2:5:3 respectively, and X-tremeGENE HP DNA Transfection Reagent (Roche, Switzerland). Cells were incubated at 37°C, 5% CO_2_ in complete DMEM for 16 hours, after which time the media was replaced with fresh complete DMEM containing HEPES 10 mM, pH 7.4. Viruses were harvested after 24 hours, filtered, and added to THP-1 cells in the presence of 8 μg/mL polybrene. THP-1 cells were then cultured for 4 weeks with 5 μg/mL puromycin to select for transductants.

### Flow cytometric analysis

Fluorescent anti-FcγR antibodies for FcγR detection were diluted in phosphate-buffered saline (PBS) 1% BSA solution and incubated with cells for 30 minutes on ice. Cells were then washed two times with PBS and analyzed by flow cytometry using a BD LSRFortessa X-20 (Beckton Dickson, USA). Similarly, a fluorescent anti-human CD14 antibody was used to detect the expression of this molecule on the cell surface. Data analysis was performed using FlowJo v10 (Beckton Dickson, USA).

### Antibody deglycosylation

Antibodies 3G8, 10.1, AT10 and IV.3 (0.5 mg/mL) in PBS without calcium and magnesium (Gibco, USA) were fully deglycosylated using 8 units/μL of glycerol-free recombinant PNGase-F (NEB, USA) and incubated for 48 hours at 37°C, 5% CO_2_. The glycans and PNGase F were removed from the antibody sample by using a 50 kDa molecular weight cut-off column concentrator (Millipore Sigma, Canada) with repeated washing with PBS.

### Phagocytosis of antibody-opsonized erythrocytes

THP-1 or THP-1-CD16A cells were seeded into wells of 24-well polystyrene plates (Corning Incorporated, USA) at 2x10^5^ cells/well in complete RPMI medium also containing 100 ng/mL of phorbol 12-myristate 13-acetate (PMA) (BioShop, Canada) for macrophage differentiation [34], and incubated at 37°C, 5% CO_2_. Twenty-four hours later, medium was replaced by fresh complete RPMI medium, and cells were maintained under the same conditions overnight. RhD^+^ human erythrocytes were washed three times with PBS by centrifugation at 170xg for 2 minutes without brake and resuspended in 0.5 mL PBS. Erythrocyte concentration was determined using a Guava easyCyte flow cytometer (Luminex Corporation, USA) and adjusted to 5x10^8^ cells/mL. Erythrocytes were opsonized with a polyclonal anti-human RhD antibody (WinRho SDF^TM^) for 30 minutes at room temperature and washed by centrifugation. Concurrently, macrophages were treated with deglycosylated FcγR-blocking antibodies or isotype controls at a concentration of 10 μg/mL diluted in complete RPMI medium for 30 minutes at 37°C, 5% CO_2_, followed by two washes with PBS. Complete RPMI was added back to all wells, and opsonized erythrocytes were added to macrophages (1x10^7^ erythrocytes per well, 20:1 ratio erythrocyte per macrophage). Phagocytosis proceeded for 30 minutes at 37°C, 5% CO_2_, after which the reaction was stopped on ice. Wells were washed with PBS before performing a 60-second hypotonic lysis of the erythrocytes using water followed by 10X PBS to return isotonicity and a final wash with PBS. Macrophages were then fixed with 4% paraformaldehyde solution in PBS and imaged by microscopy using a Nikon Eclipse TS100 inverted microscope near the center of each well. At least four non-overlaping images were taken per well for >500 macrophages counted. Phagocytic index was calculated as:

[(Total amount of erythrocytes internalized) / (Total amount of macrophages counted)] x 100

### Phagocytosis of antibody-opsonized platelets

THP-1-CD16A cells were seeded on sterile glass coverslips (Thermo Fisher Scientific, USA) in a 24-well polystyrene plate at a concentration of 2x10^5^ cells/well in complete RPMI and incubated overnight at 37°C, 5% CO_2_. PMA-induced differentiation of THP-1-CD16A cells was performed as described in the previous section. The following day, healthy human donor whole blood was collected into a tube containing anticoagulant citrate-dextrose solution (BD, USA). Platelet-rich plasma was collected by centrifugation (400xg, 8 minutes, brake set to low) and platelets were counted using a Z2 Coulter Counter (Beckman Coulter, USA). Platelets were kept in the presence of 100 ng/mL of Prostaglandin E1 (Sigma-Aldrich, USA) for all manipulations to prevent activation. PBS-EDTA 0.1% was added, and platelets were centrifuged to remove plasma (800xg, 10 minutes, brake set to low). Platelets were resuspended to 4x10^8^ platelets/mL in PBS and fluorescently labelled with 20 μM 5-chloromethylfuorescein diacetate (CMFDA) (Thermo Fisher Scientific, USA). Platelets were then incubated for 45 minutes at room temperature under constant, gentle agitation and protected from light. Platelets were washed by centrifugation (800xg, 10 minutes) and opsonized with the monoclonal antibody A2A9/6 [35] (5 μg/mL) or normal human serum (1:1 volume ratio of serum:platelet) for 30 minutes at room temperature. Platelets were washed by centrifugation and resuspended in PBS before addition to macrophages. Macrophages were treated with FcγR-blocking antibodies or isotype controls at a concentration of 10 μg/mL in complete RPMI for 30 minutes at 37°C, 5% CO_2_, and washed two times with PBS. Antibody-opsonized platelets were added to the macrophages at a ratio of 100:1 (platelets:macrophage) in complete RPMI. Phagocytosis proceeded for 60 minutes at 37°C before stopping the reaction on ice, which was followed by a wash with PBS and paraformaldehyde fixation (4% solution in PBS). After fixation, wells were washed with PBS and surface-bound platelets were stained for 30 minutes with an Alexa Fluor 647-conjugated mouse anti-human CD42a to help discern surface-bound (non-phagocytosed) platelets from phagocytosed platelets. Wells were washed two times with PBS. The coverslips were mounted onto glass slides (Thermo Fisher Scientific, USA) with Dako Fluorescence Mounting Medium (Agilent Technologies, USA). Macrophages were observed by spinning-disc confocal microscopy under 63x objective oil immersion (numerical aperture 1.47) with differential interference contrast (DIC) and laser fluorescence (488 nm, 647 nm excitation) on a Quorum multi-modal imaging system (Quorum Technologies, Canada) equipped with 50 micrometer pinhole spinning disc and ORCA-Flash 4.0 V2 PLUS sCMOS camera. At least four different images were taken near the center of each well for >500 macrophages imaged, with Z-stacking every 0.33 μm with >30 stacks. Z-stacked images were 3D reconstructed for analysis using Imaris v8.0.2 (Bitplane, UK). Surface-bound platelets were distinguished from phagocytosed platelets by 3D reconstruction and Alexa Fluor 647 staining. Phagocytic index was calculated as:

[(Total amount of platelets internalized) / (Total amount of macrophages counted)] x 100

### Statistical analysis

Prism version 8.00 for Windows (GraphPad Software, San Diego, CA, USA) was used for statistical analysis. Data normality was verified using D’Agostino-Pearson’s test, and homogeneity of variance was checked using Bartlett’s test. Non-parametric tests were used for further analysis whenever data was not normally distributed even after transformation. Parametric analyses of more than two groups were performed using a one-way analysis of variance with a post-hoc Tukey’s test.

## Results

### FcγRIIIA-transduced THP-1 (THP-1-CD16A) cells consistently express the receptor

**Fig 1:**
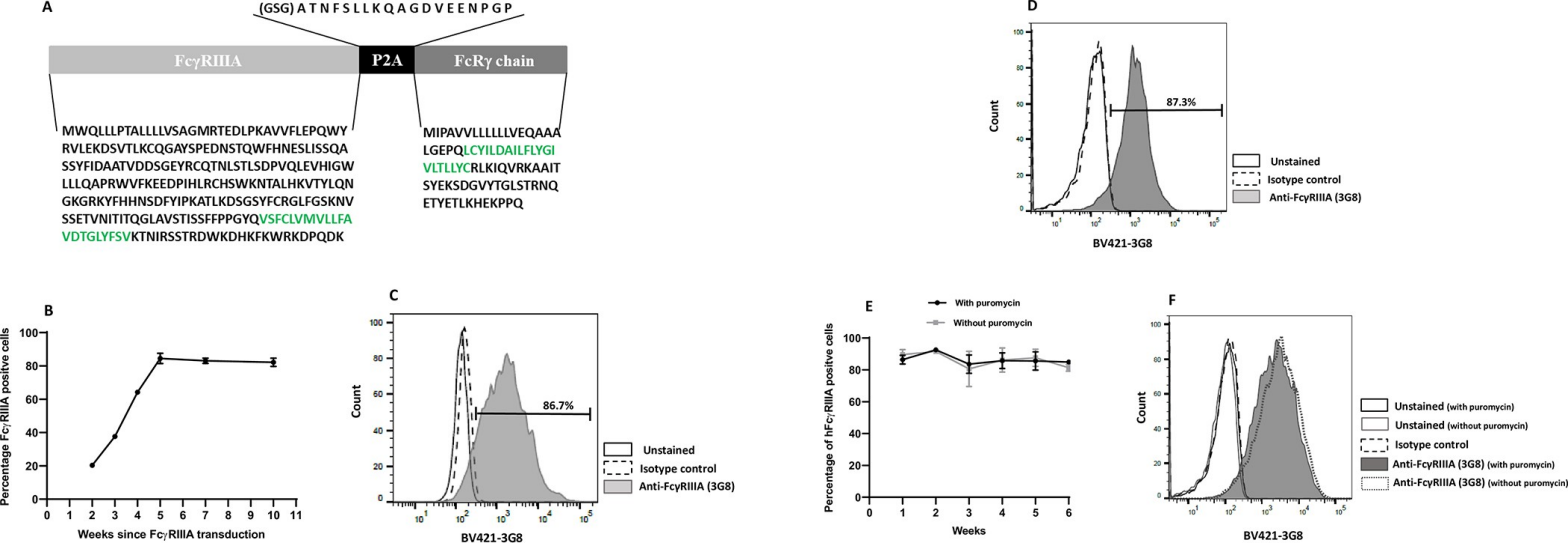
Generation of transgenic THP-1 cells expressing the low affinity FcγRIIIA and the FcRγ chain (THP-1-CD16A cells). (A) Amino acid sequence of the FcγRIIIA-FcRγ chain transgene inserted by lentiviral transduction to obtain long-term expression of FcγRIIIA. The amino acids highlighted in green represent the transmembrane region. The sequence encoding peptide 2A (P2A) was used to generate a bicistronic vector driving equimolar expression of both proteins from the same mRNA. (GSG) residues were added to the 5’-end of P2A to improve cleavage efficiency. The lentiviral system also included a puromycin-resistance gene for the selection of transduced cells. (B) Puromycin selection of lentiviral transduced THP-1 cells. THP-1 cells, following FcγRIIIA-FcRγ chain lentiviral transduction, were cultured in the presence of puromycin (5 μg/mL) to select against non-transduced THP-1 cells. FcγRIIIA expression was assessed by flow cytometry on weeks 2, 3, 4, 5, 7 and 10 after transduction. FcγRIIIA expression was detected using a BV421-conjugated mouse anti-human FcγRIIIA (clone 3G8) antibody. Data are presented as the mean ± the standard deviation from two independent determinations. (C) The histogram represents one of the measurements performed on week 5. (D) The histogram represents a measurement after 40 weeks of cell culture with different numbers of passages in puromycin. (E) THP-1-CD16A cells were cultured with or without puromycin, and the expression of FcγRIIIA was evaluated using the BV421-conjugated mouse anti-human FcγRIIIA (clone 3G8) antibody for 6 weeks. Data are presented as the mean ± the standard deviation from two independent experiments. (F) The histogram represents one of the measurements performed on week 6. Flow cytometry was performed using a BD LSRFortessa X-20. Data analysis was performed using FlowJo v10.

The human FcγRIIIA utilizes an ITAM that is located on the FcRγ chain [1]. To obtain a lentivirus system that could fully transduce the THP-1 cells with the human FcγRIIIA, we first amplified the sequence that encoded the FcγRIIIA (CD16F158) and FcRγ chain by PCR. FcγRIIIA and FcRγ chain PCR products were then cloned into the KpnI and SmaI sites respectively, of the pUC19 plasmid [36]. This construction was further used as a template to introduce a picornavirus sequence encoding for 2A self-cleaving peptides (P2A) between the sequences that encode for the FcγRIIIA and FcRγ chain using a fast-cloning PCR procedure [32] (Fig 1A). The genetic sequence that encodes for the FcγRIIIA-P2A-FcRγ (Fig 1A) was then cloned into the lentiviral vector pVLX-puro that contains a puromycin-resistance gene.

HEK-293T/17 cells were then transfected with the lentiviral system, as described in the Material and Methods section. Forty hours later, the supernatant was collected, and cells were analyzed by flow cytometry. Approximately 80% of transfected cells expressed the human FcγRIIIA (S1A and S1B Fig) demonstrating the efficiency of the designed lentiviral system. The HEK293T cells were able to bind to erythrocytes coated with an anti-human RhD antibody, and this binding was inhibited by an FcγRIIIA blocking antibody. However, the cells were unable to meaningfully phagocytose the erythrocytes (S1C and S1D Fig), suggesting that the mere expression of FcγRIIIA is not sufficient for phagocytosis. Harvested lentiviruses were then used to infect THP-1 cells, and the cells were cultured in the presence of 5 μg/mL puromycin to select for the successfully transduced cells. Two weeks after the infection, 21.1% of the cells expressed the receptor on their surface, which increased to 65% two weeks later (Fig 1B). From week 5 to week 10, over 85% of the cells showed the expression of the receptor (Fig 1B and 1C). The percentage of FcγRIIIA-positive cells was observed for over 40 weeks of cell culture with different number of passages in puromycin (Fig 1D). In addition, the expression of this receptor was stable for at least 6 weeks of cell culture without puromycin (Fig 1E and 1F).

### The transduction of THP-1 cells with FcγRIIIA does not affect the expression of endogenous FcγRs

**Fig 2:**
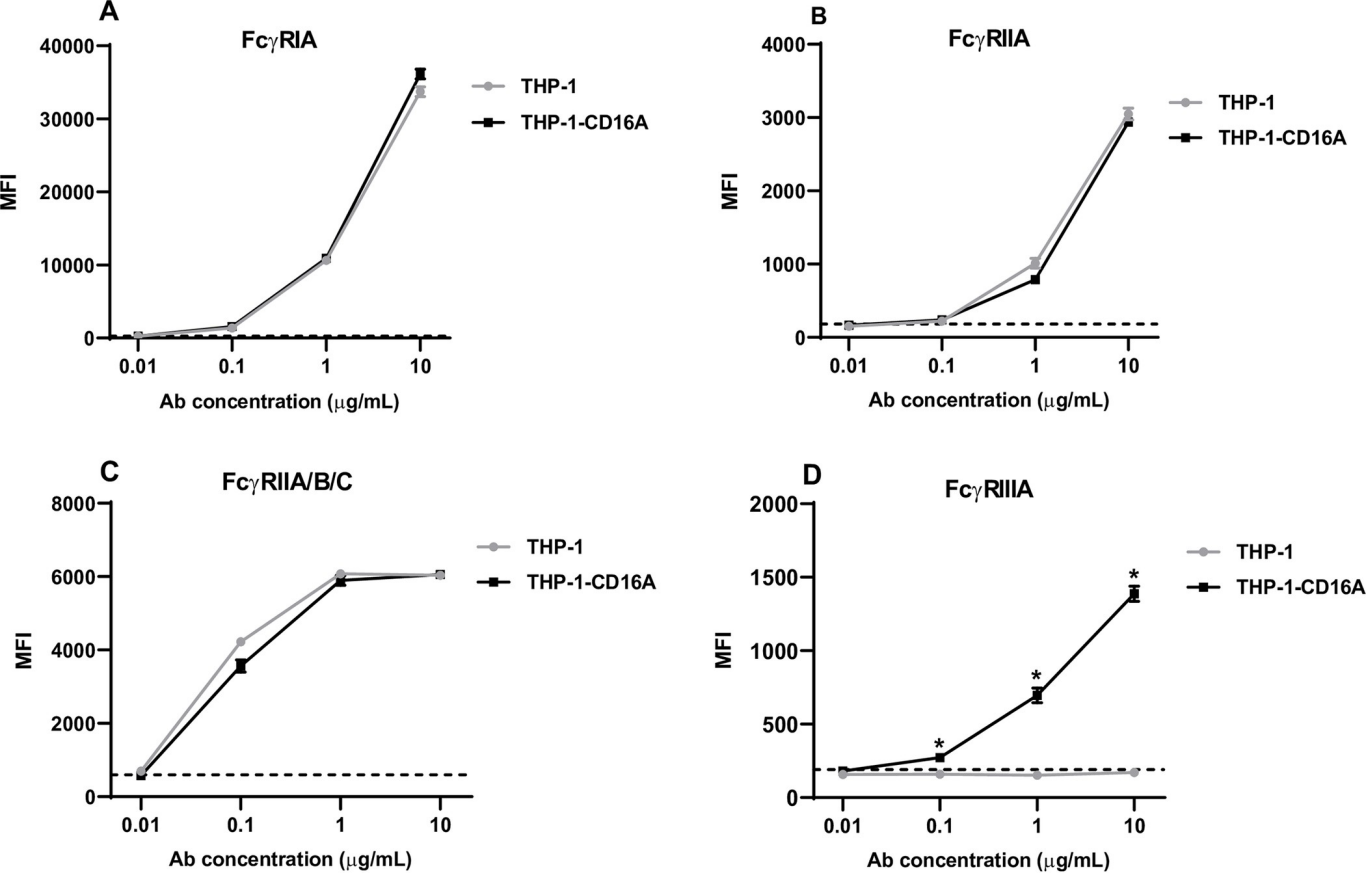
Expression of human FcγRs on THP-1 and THP-1-CD16A cells. Five hundred thousand cells were stained with direct-labelled monoclonal antibodies against (A) FcγRI (clone 10.1 conjugated to PE/Cy7), (B) FcγRIIA (clone IV.3 conjugated to FITC), (C) FcγRIIA/B/C (clone AT10 conjugated to AF647), and (D) FcγRIIIA (clone 3G8 conjugated to BV421). Stained cells were washed and analyzed by flow cytometry using a BD LSRFortessa X-20. Data analysis was performed using FlowJo v10. Data are presented as the mean ± the standard deviation from four independent experiments. MFI: Mean fluorescent intensity (arbitrary units). Antibody concentration: Concentration of the fluorescent antibody for FcγR expression detection. The dashed line represents the MFI value for the corresponding isotype control at 10 μg/mL. The statistical analysis was performed using the non-parametric Mann-Whitney T-test, comparing the MFI of both cell lines for each antibody concentration (*: *p* = 0.05).

Once the expression of the human FcγRIIIA on THP-1 cells (THP-1-CD16A cells) was stabilized overtime, the endogenous expression of other human FcγRs was analyzed by flow cytometry. Two antibodies were used to detect the expression of FcγRII: clone IV.3 that recognizes FcγRIIA and clone AT10 for the recognition of all FcγRII isoforms (FcγRIIA/B/C). Expression levels of all FcγRs in THP-1-CD16A cells were comparable the wild type THP-1 cells (Fig 2A–2C), except for FcγRIIIA, which was only expressed on the transduced cells (Fig 2D). No statistical differences were detected for the expression of FcγRI, FcγRIIA, and FcγRII isoforms when both cell lines were compared (*p*>0.05).

### Differentiation of THP-1-CD16A cells to macrophages increases the expression of FcγRIIIA and reduces the expression of FcγRIA and FcγRII

THP-1 cells have a monocytic phenotype and they can be differentiated into macrophages that resemble human monocyte-derived macrophages [27]. Here, we investigated whether the new THP-1-CD16A cell line retained the expression of various FcγRs after differentiation into macrophages with PMA.

**Fig 3:**
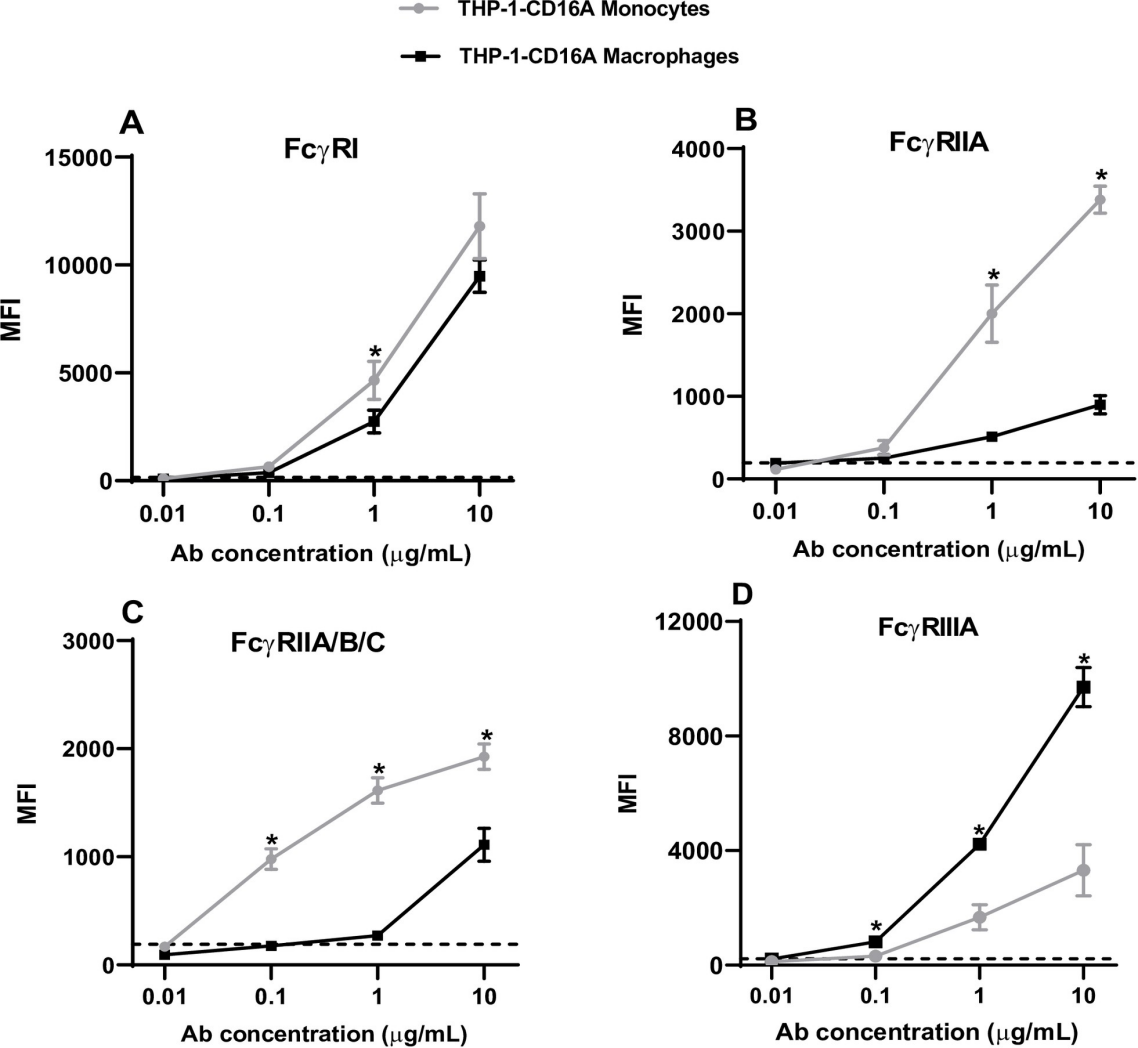
Expression of human FcγRs on non-differentiated and PMA-differentiated THP-1-CD16A cells. THP-1-CD16A cells were differentiated to macrophages by treatment with PMA (100 ng/mL) as described in the Materials and methods. Five hundred thousand cells were stained with direct-labelled monoclonal antibodies against (A) FcγRI (clone 10.1 conjugated to PE/Cy7), (B) FcγRIIA (clone IV.3 conjugated to FITC), (C) FcγRIIA/B/C (clone AT10 conjugated to AF647), or (D) FcγRIIIA (clone 3G8 conjugated to BV421). Stained cells were washed and analyzed by flow cytometry using a BD LSRFortessa X-20. Data analysis was performed using FlowJo v10. Data are presented as the mean ± the standard deviation from four independent experiments. MFI: Mean fluorescent intensity (arbitrary units). Ab concentration: Concentration of the fluorescent antibody for FcγR expression detection. The dashed line represents the MFI value of the corresponding isotype control at 10 μg/mL. The statistical analysis was performed by the non-parametric Mann-Whitney T-test, comparing the MFI of both conditions for each antibody concentration (*: *p* = 0.05).

Flow cytometric analysis of PMA-differentiated THP-1-CD16A cells to macrophages showed an increased size (FSC-A), granularity (SSC-A), and CD14 expression (S2 Fig). This expression pattern has been previously reported for wild type THP-1 cells [27]. In comparison with non-differentiated THP-1-CD16A cells (monocytes), a slight reduction in the expression of FcγRI (Fig 3A) was observed in the differentiated cells (*p*<0.05) whereas the expression of the FcγRIIA and FcγRII(A/B/C) isoforms (Fig 3B and 3C) were reduced more dramatically (*p*<0.05). On the contrary, expression of the transduced receptor (FcγRIIIA) was dramatically increased in PMA-differentiated macrophages (Fig 3D). A similar overall behavior was detected when we analyzed the expression of FcγRs on THP-1 parental cells (monocytes and macrophages) except for the FcγRIIIA, which is not expressed on wild type THP-1 monocytes or PMA-differentiated macrophages (S3 Fig).

### FcγRIIIA on THP-1-CD16A cells contributes to the phagocytosis of IgG-opsonized human erythrocytes and platelets

**Fig 4:**
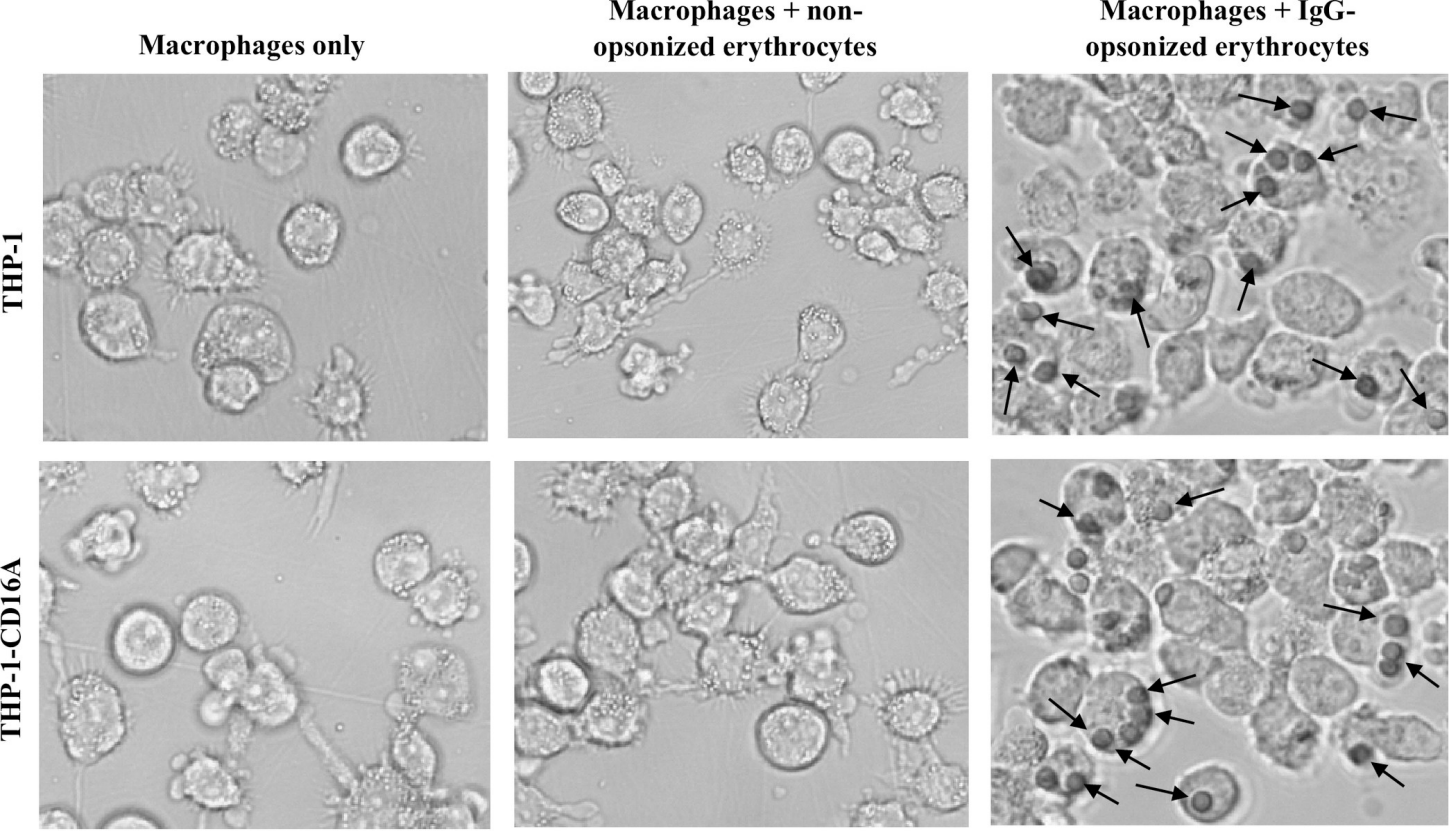
Brightfield micrographs of THP-1 or THP-1-CD16A macrophages demonstrating phagocytosis of IgG-opsonized human erythrocytes. Macrophages were incubated with human erythrocytes previously opsonized with a polyclonal anti-human RhD antibody (WinRho SDF^TM^). External, non-phagocytosed erythrocytes were removed by hypotonic (water) lysis. Black arrows indicate examples of phagocytosed erythrocytes. Micrographs are representative of five independent experiments.

**Fig 5:**
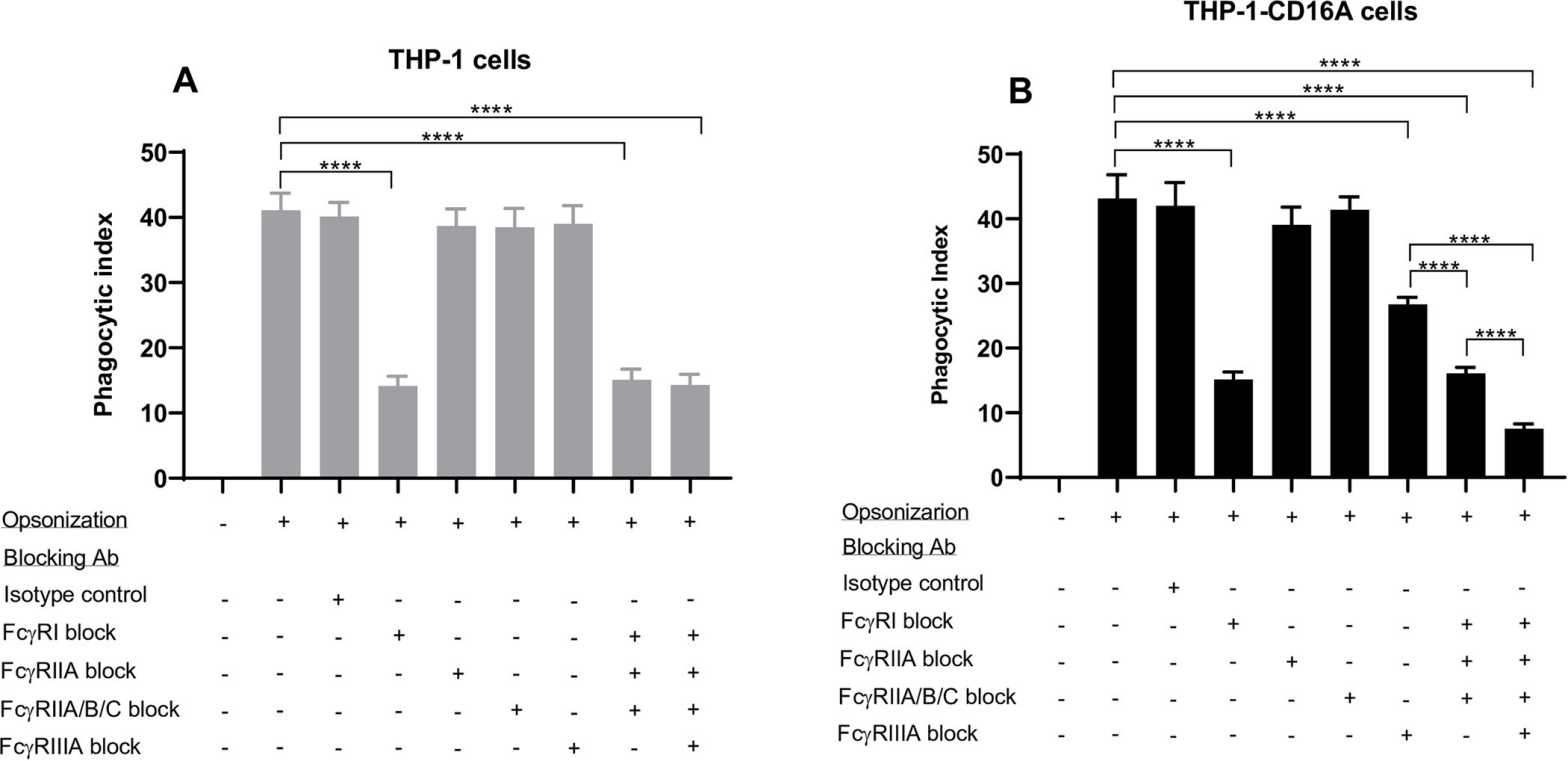
FcγR utilization by THP-1 and THP-1-CD16A cells in the phagocytosis of IgG-opsonized human erythrocytes. THP-1 cells were differentiated to macrophages by treatment with PMA (100 ng/mL) as described in the methods. (A) THP-1 cells. (B) THP1-CD16A cells. Opsonization: (-) indicates erythrocytes were non-opsonized (incubated with phosphate buffered saline), (+) indicates erythrocytes were opsonized with a polyclonal anti-human RhD antibody (WinRho SDF^TM^). The contribution of each FcγR to the phagocytosis was evaluated using Fc region deglycosylated blocking antibodies (final concentration of 10 μg/mL each): anti-FcγRI (clone 10.1), anti-FcγRIIA (clone IV.3), anti-FcγRIIA/B/C (clone AT10), or anti-FcγRIIIA (clone 3G8). The deglycosylated mouse IgG1 (clone MOPC-21) and deglycosylated mouse IgG2b (clone MPC-11) were used in combination as isotype controls (final concentration of 10 μg/mL each). The phagocytic index was calculated as the number of erythrocytes engulfed per 100 macrophages. Data are presented as the mean ± the standard deviation of five independent experiments. For panels A and B, the statistical analysis was performed with a one-way analysis of variance (ANOVA) and Tukey’s multiple comparisons test (****: *p* <0.001).

To explore the functionality of the transduced cells, we evaluated their capability to phagocytose IgG-opsonized human erythrocytes. Previous studies have demonstrated the phagocytic capacity of THP-1 cells [27]. Therefore, wild type THP-1 cells were used as a reference control in these experiments. Cells were incubated with non-opsonized or RhD^+^ red blood cells (RBCs) opsonized with anti-D, a commercial human polyclonal preparation of purified IgG with specificity for the cell surface RhD antigen. Both THP-1 and THP-1-CD16A cells were able to phagocytose the human RBC when they were opsonized with anti-D (Fig 4), and no differences in phagocytic index were observed between the cell types (Fig 5).

We then evaluated the utilization of each FcγR in the phagocytic activity of the THP-1-CD16A or wild type THP-1 cell line. Cells were treated with FcγR-specific blocking antibodies or isotypes controls. All blocking antibodies were deglycosylated to reduce non-specific blockade. The deglycosylation was evaluated by SDS-PAGE as shown in S4 Fig, which demonstrated the expected decrease in the molecular weight (⁓3 KDa) of each antibody heavy chain. Blocking FcγRI inhibited approximately 75% of the phagocytosis in both cell lines (Fig 5A and 5B), demonstrating that FcγRI maximally contributed to the phagocytosis of anti-D-opsonized RBCs. For both cell lines, the blockade of FcγRIIA or FcγRII isoforms did not meaningfully affect the phagocytosis of the anti-D-opsonized RBCs. In wild type THP-1 cells a similar phagocytic index was observed when FcγRIIIA was blocked compared to cells incubated without any blocking antibody (Fig 5A). However, in THP-1-CD16A cells, roughly 30% of phagocytosis was reduced when they were incubated with a FcγRIIIA blocking antibody (Fig 5B), demonstrating the contribution of FcγRIIIA in the phagocytosis of anti-D-opsonized human erythrocytes in these cells. Moreover, a very low level of phagocytosis was attained when THP-1-CD16A cells were treated with a combination of all blocking antibodies (Fig 5B), demonstrating that multiple FcγRs were involved in the phagocytosis of the RBC. As expected, the contribution of FcγRIIIA was not apparent upon the combination of the blocking antibodies in wild type THP-1 cells (Fig 5A).

**Fig 6:**
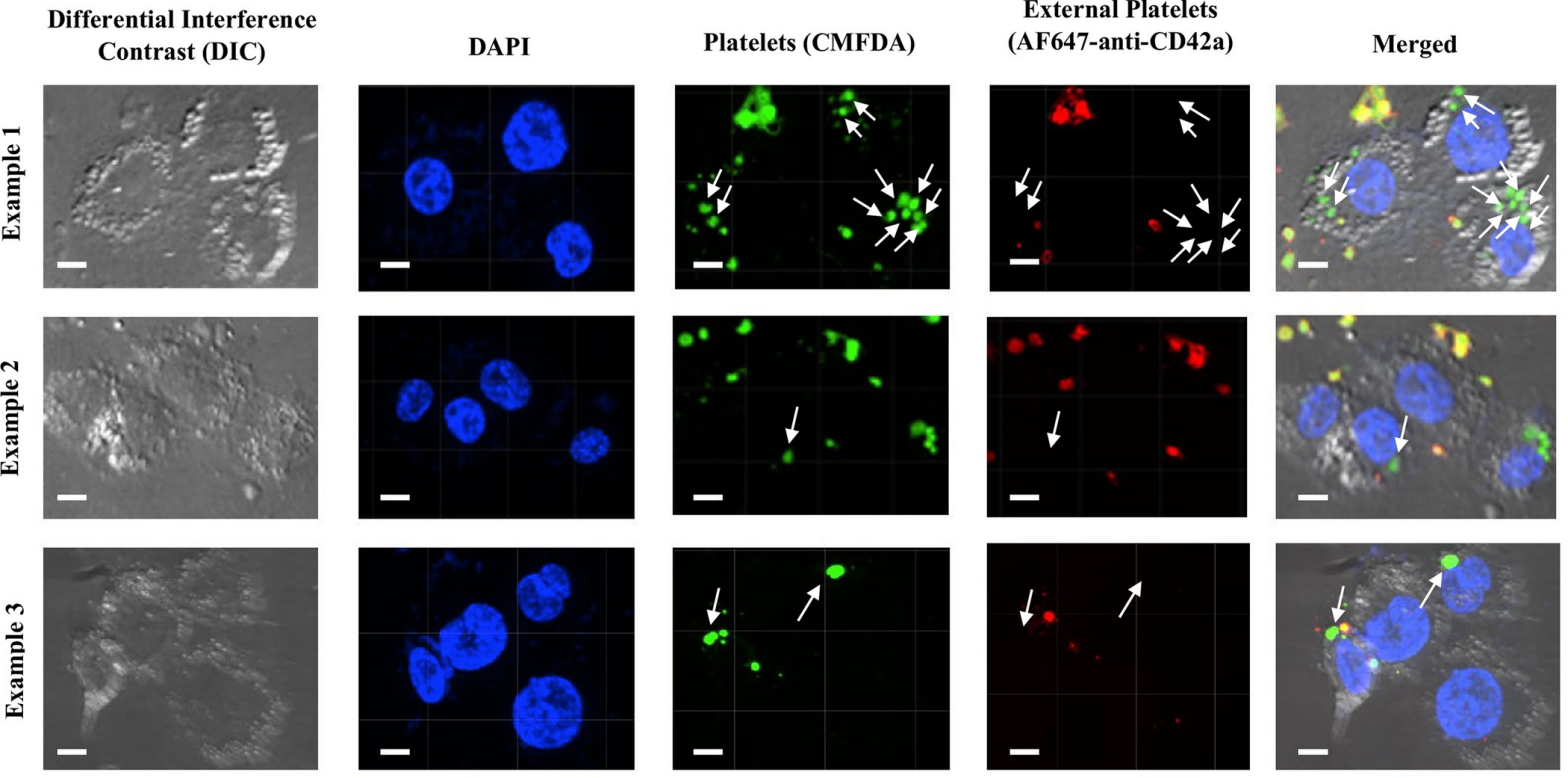
THP-1-CD16A macrophages with phagocytosed IgG-opsonized human platelets. Three different images are shown of IgG-opsonized platelets incubated with THP-1-CD16A macrophages. Platelets were labelled with 5- chloromethylfluorescein diacetate (CMFDA) (green) before phagocytosis. Non-phagocytosed platelets were differentiated after phagocytosis using an AF647-conjugated anti-human CD42a antibody (red). Platelets were additionally defined by size (roughly 1.5 to 3.5 μm) to distinguish them from internalized microparticles or platelet aggregates. THP-1-CD16A macrophages were observed by spinning-disc confocal microscopy under 63x objective oil immersion with differential interference contrast and DAPI stain on a Quorum multi-modal imaging system (Quorum Technologies, Ontario, Canada). Four images were taken at the center of each well with Z-stacking. Phagocytosis was quantified using Imaris v9.6.0. Scale bar = 3 μm. White arrows indicate examples of phagocytosed platelets.

**Fig 7:**
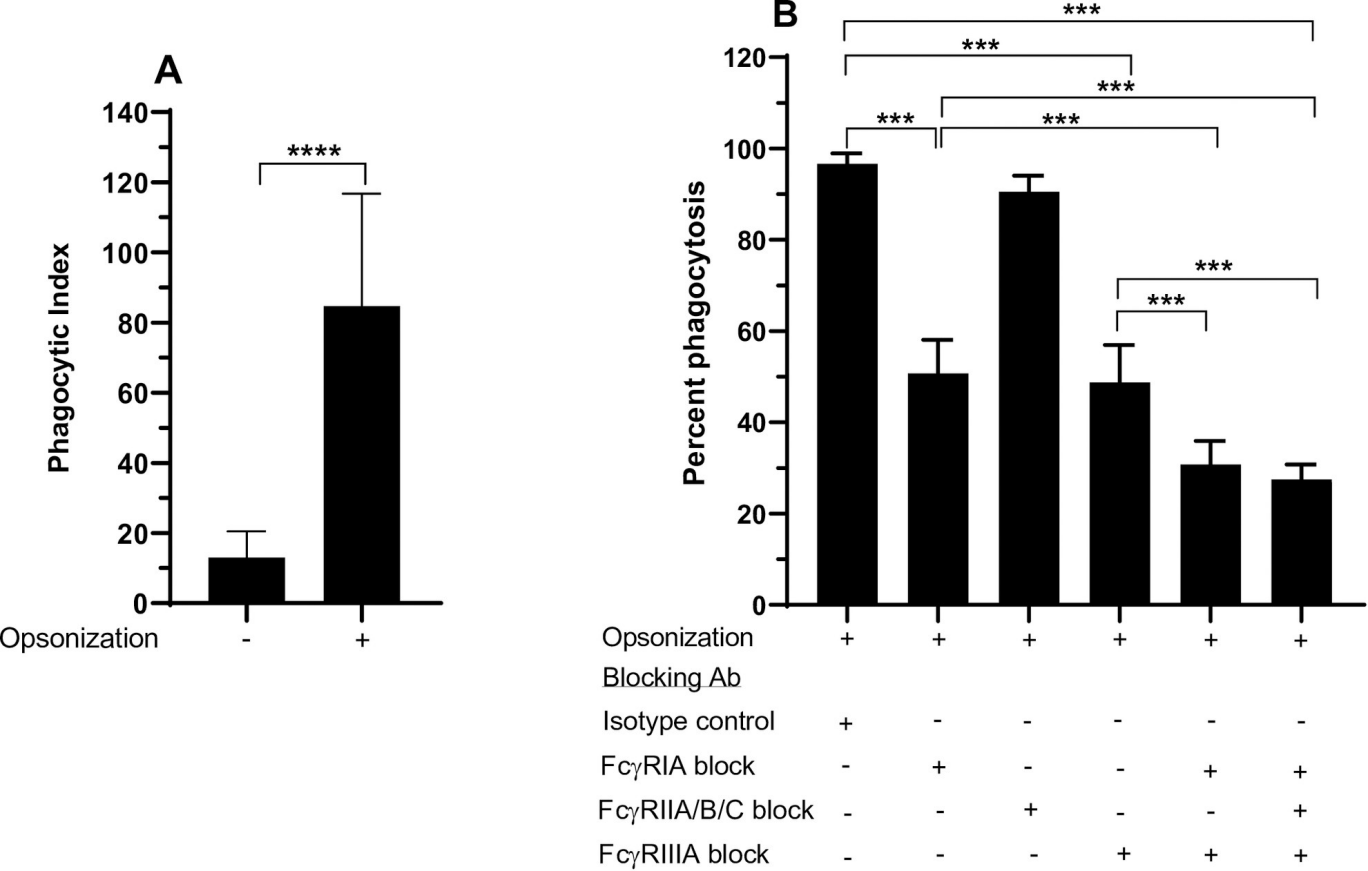
FcγR utilization by THP-1-CD16A cells in the phagocytosis of IgG-opsonized human platelets. THP-1-CD16A cells were differentiated to macrophages by treatment with PMA (100 ng/mL) as described in the Materials and methods. (A) Magnitude of macrophage phagocytosis of IgG-opsonized platelets. Opsonization: (-) indicates platelets were non-opsonized (incubated with normal human serum), (+) indicates platelets were opsonized with the monoclonal antibody A2A9 (5 μg/mL). The phagocytic index was calculated as the number of platelets engulfed per 100 macrophages. Data are presented as the mean ± the standard deviation of eight independent experiments. The statistical analysis was performed by the non-parametric Mann-Whitney T-test (****: p <0.0001). (B) The contribution of each FcγR to the phagocytosis was evaluated using Fc region deglycosylated blocking antibodies (final concentration of 10 μg/mL each): anti-FcγRI (clone 10.1), anti-FcγRIIA/B/C (clone AT10), or anti-FcγRIIIA (clone 3G8). The deglycosylated mouse IgG1 (clone MOPC-21) was used as isotype control (final concentration of 10 μg/mL). Percent phagocytosis was assessed relative to macrophages without any antibody treatment of the THP-1-CD16A cells. Data are presented as the mean ± the standard deviation of five independent experiments. The statistical analysis was performed with a one-way analysis of variance (ANOVA) and Tukey’s multiple comparisons test (***: *p* <0.001).

To further evaluate FcγR functionality in THP-1-CD16 cells, human platelets were sensitized with the antiplatelet mouse IgG2a antibody A2A9/6 [35], which recognizes the cell surface glycoprotein IIb/IIIa. THP-1-CD16A macrophages were able to phagocytose IgG-opsonized platelets (Figs 6 and 7A). The macrophages were then treated with antibodies that blocked different FcγRs classes, which demonstrated that FcγRI and FcγRIIIA equally contributed to the phagocytosis of IgG-sensitized platelets with no apparent contribution of the FcγRII isoforms (Fig 7B). An additive effect on the blockade of phagocytosis was observed when macrophages were treated with a mixture of FcγRI and FcγRIIIA blocking antibodies (Fig 7B). A similar level of phagocytosis was observed when we added a FcγRII blocking antibody to this mixture.

## Discussion

FcγRs are critical effector receptors for IgG antibodies [3,7]. On macrophages, FcγRs mediate multiple effector functions such as phagocytosis, ADCC, and antibody-dependent inflammation [6,37]. Consequently, macrophage FcγRs are associated with protection against infectious diseases but also pathogenicity in several antibody-mediated inflammatory disorders.

Both primary macrophage cell cultures and cell lines have been used extensively to study the properties and effector functions of FcγRs on macrophages [31]. While primary macrophage sources such as bone marrow-derived macrophages, monocyte-derived macrophages, or splenic macrophages may theoretically better reflect endogenous macrophage biology, they can be limited by the ability to collect a sufficient number of primary cells, variability between donors, and the presence of contaminant cells [31]. With respect to human primary cell studies, peripheral blood monocytes are typically the most common source of cells for macrophage generation, given the obvious limitations in studying human bone marrow-derived or splenic macrophages. In addition, there are three different subsets of monocytes in peripheral blood: classical, intermediate, and nonclassical monocytes. Unfortunately, classical monocytes, which represent 80–90% of these cells, do not express FcγRIIIA [28,29], an important activating receptor that can mediate phagocytosis and ADCC [38,39].

Monocyte or macrophage cell lines can overcome many of the limitations associated with primary cells. Common monocyte cell lines that can be differentiated into macrophages include THP-1, U937, and Mono-Mac-6 cells. Unfortunately, these monocyte cell lines also lack expression of FcγRIIIA [27,40–43]. Furthermore, U937 and Mono Mac 6 cells also appear to lack expression of FcγRIIA (CD32H131) and FcγRIIB, respectively [40,43]. Differentiation of THP-1 monocytes into macrophages with PMA has been found not to lead to FcγRIIIA expression [27], which we also observed in our work (S3 Fig). There is evidence that cytokines plus Toll-like receptor agonists (such as IFN-γ plus LPS) may induce FcγRIIIA expression in THP-1 cells [30]. However, in our experience, we were unable to observe FcγRIIIA expression using this method (unpublished). The lack of FcγRIIIA expression in most peripheral blood monocytes and monocyte cell lines has made studying macrophage FcγRIIIA function challenging.

To overcome limited FcγRIIIA expression in monocyte cell lines and monocyte-derived macrophages, we generated transgenic THP-1 cells constitutively expressing FcγRIIIA via lentiviral transduction. The genes encoding for human FcγRIIIA (CD16AF158) and the FcRγ chain were introduced into the lentiviral vector pVLX-puro as a bicistronic sequence, with the two genes separated by a 2A peptide (P2A) [33] element. The "self-cleaving" P2A was used instead of an internal ribosomal entry site because it allows to produce equimolar amounts of both genes from the same mRNA sequence.

HEK-293T/17 cells transfected with the lentiviral system expressed FcγRIIIA (S1 Fig) demonstrating the functionality of the described construction. However, these transfected cells were unable to phagocytose human erythrocytes opsonized with a polyclonal anti-human RhD antibody, despite reasonable surface FcγRIIIA expression and the ability to bind the opsonized RBCs (S1 Fig). This result suggests that the mere expression of FcγRs does not necessarily confer a biologically functional level of phagocytic activity. Nagarajan and colleagues reported in 1995 that Chinese hamster ovary cells transfected with human FcγRIIIA phagocytosed IgG-coated erythrocytes [44], although the level of phagocytosis appeared to be very low, and the authors only used an indirect (enzymatic) assay to detect phagocytic activity. In addition, the well-known ability of FcγRIIA to bind IgG in cell-free systems [45] did not translate into phagocytic function in either THP-1 or THP-1-CD16A macrophages, highlighting the importance of studying FcγRs in the context of cells that can normally express these receptors before drawing conclusions about function.

To select for FcγRIIIA-transduced THP-1 cells, we utilized a puromycin resistance gene encoded in the lentiviral vector. Using this method, we obtained a population of THP-1-CD16A cells with at least 85% of cells positive for FcγRIIIA expression above background threshold levels by flow cytometry. Expression of FcγRIIIA was maintained after repeated passaging in puromycin-supplemented media. The endogenous expression of the other FcγRs remained equivalent to wild-type THP-1 cells and did not appear affected by the lentiviral transduction of FcγRIIIA. Furthermore, the changes in endogenous FcγR expression following THP-1 macrophage differentiation via PMA [27] (S3 Fig) were also observed with THP-1-CD16A cells. Therefore, FcγRIIIA transduction did not appear to alter the expression of other FcγRs. Interestingly, we found that PMA stimulation up-regulated FcγRIIIA expression in THP-1-CD16A cells. To our knowledge, this is the first demonstration of FcγRIIIA expression on THP-1 cells by lentiviral transduction. Therefore, the increased expression of FcγRIIIA on THP-1-CD16A macrophages upon PMA differentiation constituted an interesting finding. Nevertheless, some authors have also described an increase in the expression of FcγRIIIA on peripheral blood mononuclear cell (PBMC)-derived macrophages in comparison to monocytes [46,47].

Given that phagocytosis is one of the main effector functions triggered by FcγRs on macrophages, the capacity of THP-1-CD16A cells to phagocytose IgG-coated targets was evaluated *in vitro*. In our study, we show that THP-1-CD16A cells successfully phagocytosed both anti-D-opsonized erythrocytes and antibody-opsonized-platelets. Critically, we observed that both FcγRI and FcγRIIIA were utilized in the phagocytosis of both targets, albeit to different extents for RBC phagocytosis. Blockade of either FcγRIIA alone or all FcγRII isoforms (FcγRIIA/B/C) did not appear to affect erythrocyte phagocytosis, perhaps informing their relevance, or lack thereof, to phagocytic activity. In the case of platelet phagocytosis, we observed an equal utilization of FcγRI and FcγRIIIA as determined by antibody blockade of these receptors. FcγRII isoforms did not appear to contribute to the phagocytosis of platelets in this system. However, it is worth noting that the monoclonal antibody used to opsonize platelets in our study (A2A9/6) is a mouse IgG2a subclass, which possesses high affinity for human FcγRI and low affinity for FcγRIIA/B/C and IIIA [48]. Therefore, it is possible that some of the phagocytosis we observed through FcγRI could be attributed to differential binding affinity. The FcγR utilization of THP-1-CD16A cells in phagocytosis appears similar to that of human splenic macrophages observed by Norris et al. (2020), who described a predominant utilization of FcγRI for anti-D-opsonized erythrocytes and a balanced utilization of FcγRI and FcγRIIIA for human platelets opsonized by serum containing anti-platelet autoantibodies [17]. However, THP-1-CD16A cells either as monocytes or macrophages are not necessarily an equivalent model of human splenic macrophages. A question left unanswered from the previous study [17] was whether the utilization of FcγRI and FcγIII for human platelet phagocytosis was specific to autoantibody-containing plasma or whether a purified monoclonal antibody can replicate this utilization. In the current study, we have utilized a monoclonal antibody to opsonize human platelets, demonstrating that purified IgG on its own is sufficient to drive human platelet phagocytosis through FcγRI and FcγIII.

FcγRI has been described as an important receptor for mediating phagocytosis [49,50], as demonstrated by mice that lack FcγRI exhibiting severely blunted macrophage phagocytic function [49]. FcγRIIIA is also considered to be a critical activating receptor involved in phagocytosis [38,51] and our work supports this conclusion. One interesting aspect of our study is that some consider FcγRIIA to be sufficient for triggering phagocytosis [23] while in other contexts, FcγRIIA has not been recognized for mediating phagocytosis, at least *in vivo* [52,53]. There was a report demonstrating that FcγRIIA-bearing COS-1 cells can phagocytose IgG-opsonized erythrocytes [54], but the level of phagocytosis appeared to be quite minimal. Similarly, we have found that HEK-293T17 cells transfected with FcγRIIIA triggered only minimal phagocytosis. Further work will be needed to confirm if nonprofessional phagocytic cells can indeed meaningfully phagocytose antibody-opsonized target cells.

In terms of the contribution of FcγRIIA to phagocytosis, Rijkers and colleagues have shown that human leukocyte antigen (HLA) antibodies capable of cross-linking HLA class I antigens and FcγRIIA on platelets can trigger platelet activation and subsequent phagocytosis of the platelets by macrophages [55]. However, this mechanism is distinct from an anti-platelet antibody engaging FcγRIIA on the surface of a macrophage. FcγRIIA has been typically shown to be associated with cytokine release, antibody-dependent inflammation, and antibody-dependent enhancement of infection [53,56]. One caveat of our study is that we did not evaluate the role of FcγRIIB to phagocytosis. It is possible that after engagement with an IgG opsonized target, FcγRIIB may cross-inhibit other activating FcγRs, such as FcγRIIA. It may be of interest to perform the specific blockade or inhibition of FcγRIIB in future studies to determine if FcγRIIB may be influencing the pattern of FcγR utilization.

To date, the study of monocyte and macrophage FcγR function has been limited due to a lack of readily available monocyte/macrophages cell lines expressing FcγRIIIA. Thus, our study shows that THP-1-CD16A cells (ATCC Accession Number CRL3575) may provide a valuable and convenient tool for further studies of macrophage FcγRIIIA and other FcγR effector functions.

## Supporting information

S1 Fig Analysis of FcγRIIIA transfection in HEK293T cells. (DOCX)

S2 Fig Flow cytometric analysis of size, granularity and CD14 expression on THP-1-CD16A. (DOCX)

S3 Fig Expression of human FcγRs on non-differentiated and PMA-differentiated THP-1 cells. (DOCX)

S4 Fig SDS-PAGE analysis of blocking antibodies deglycosylation. (DOCX)
