# Supplementary material for: THP-1 cells transduced with CD16A utilize Fcγ receptor I and III in the phagocytosis of IgG-sensitized human erythrocytes and platelets
Source: PLoS One. 2022 Dec 14;17(12):e0278365. doi: 10.1371/journal.pone.0278365 (PMC9749970; doi:10.1371/journal.pone.0278365)
Supplement: S1 Fig — (DOCX) [file pone.0278365.s001.docx]

**BV421-3G8**

**Count**

**Unstained**

**Isotype control**

**Anti-FcγRIIIA (3G8)**

**B**

**79.1%**

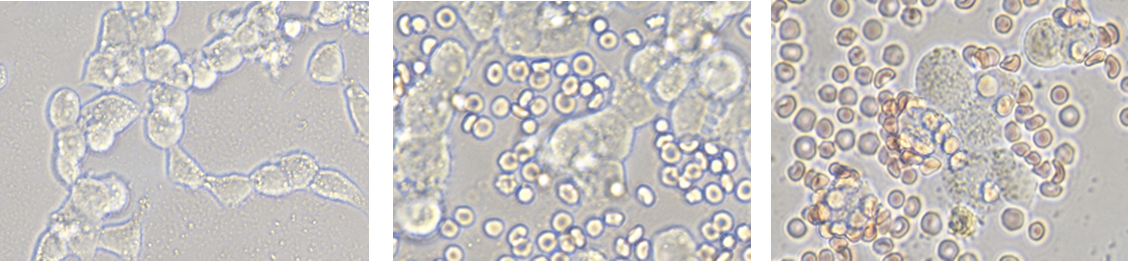


**HEK293T cells + non-opsonized erythrocytes**

**HEK293T cells only**

**HEK293T cells + IgG-opsonized erythrocytes**

**E**

**Without hypotonic lysis**


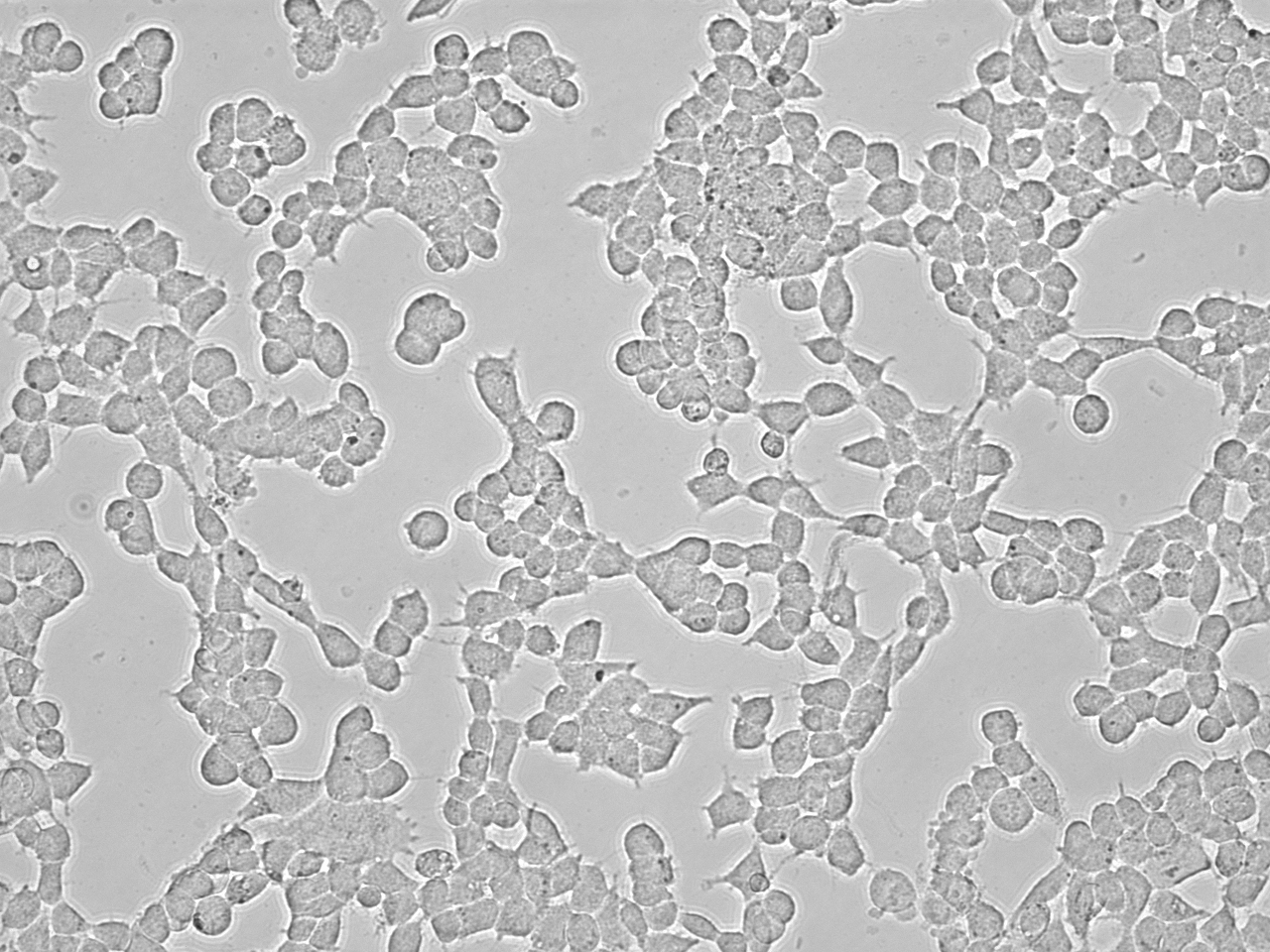

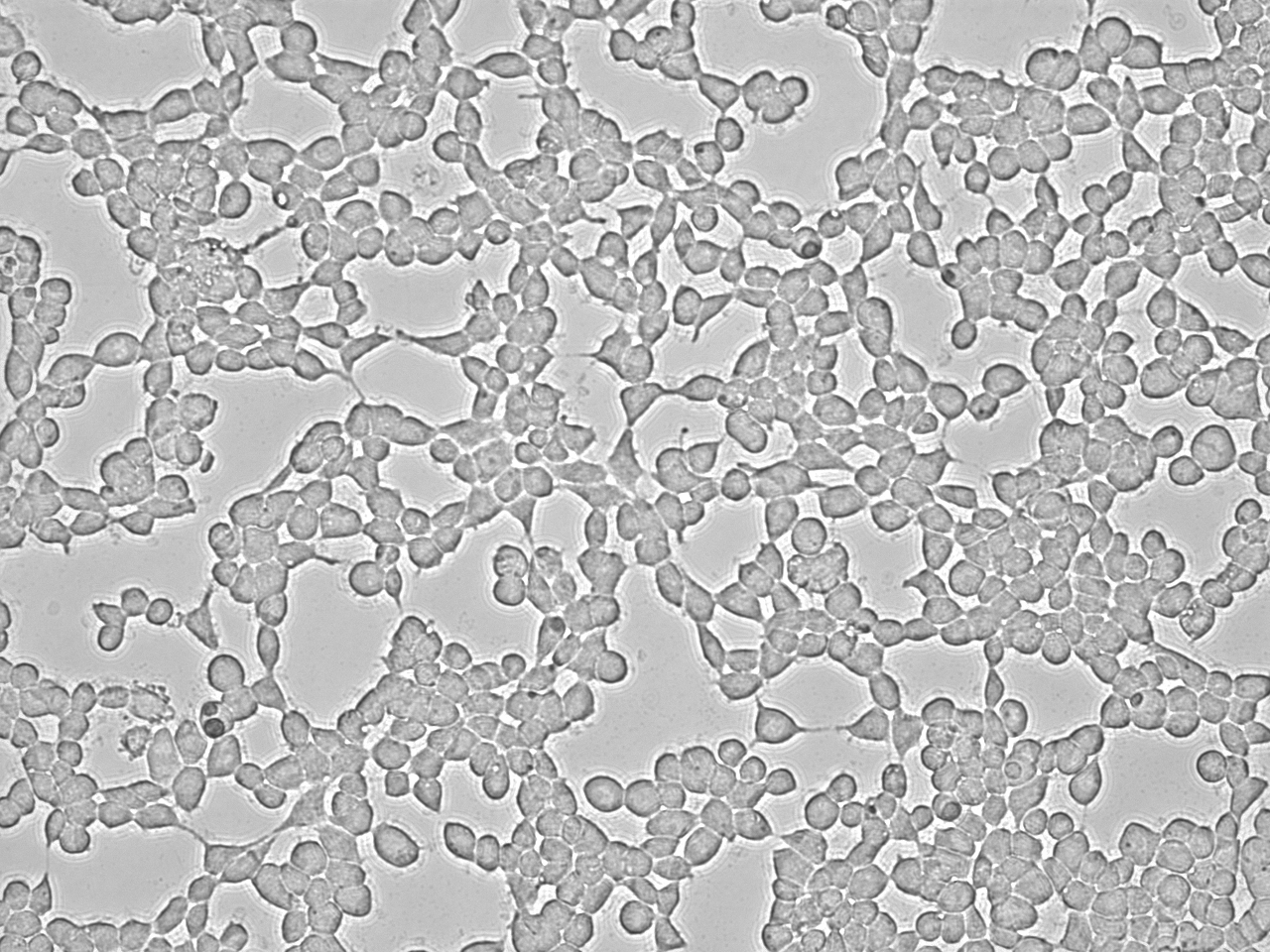

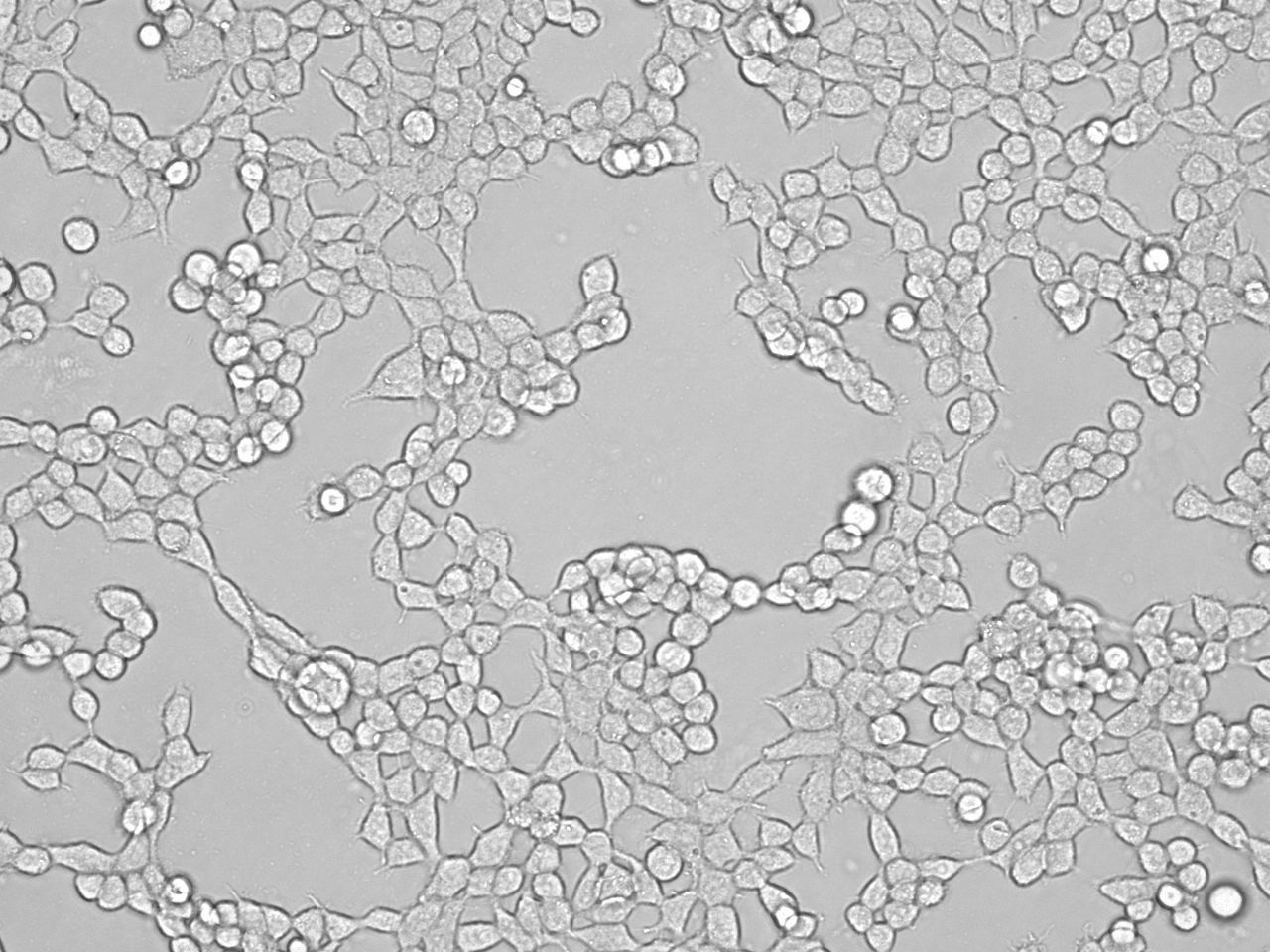


**HEK293T cells + non-opsonized erythrocytes**

**HEK293T cells + IgG-opsonized erythrocytes**

**HEK293T cells only**

**F**

**With hypotonic lysis**

**S1 Fig.** **Analysis of** **FcγRIIIA transfection in HEK293T cells.**

Cells were transfected with the plasmids pMD2-G, psPAX2 and pVLX-puro- FcγRIIIA-P2A-FcRγ for the expression of FcγRIIIA. Forty hours later, cells were analyzed for the expression of the receptor and their capacity to bind and phagocytose IgG-opsonized human erythrocytes (A) Cells were stained with a direct-labelled monoclonal antibody against FcγRIIIA (clone 3G8 conjugated to BV421). Stained cells were washed and analyzed by flow cytometry using a BD LSRFortessa X-20 from two independent experiments. MFI: mean fluorescent intensity (arbitrary units). Ab concentration: concentration of the fluorescent antibody for FcγR expression detection. The dashed line represents the MFI value for the corresponding isotype control at 10 µg/mL. (B) Histogram representation of FcγRIIIA expression using the optimal dose of the antibody (1 µg/mL). (C) To evaluate the capacity of the receptor to bind the immune complexes, FcγRIIIA-transfected HEK293T cells were incubated with human erythrocytes opsonized with a polyclonal anti-human RhD antibody. The binding index was calculated as the number of erythrocytes bound per 100 HEK293T cells. (D**)** FcγRIIIA-transfected HEK293T cells were incubated with human erythrocytes opsonized with a polyclonal anti-human RhD antibody to evaluate the capacity of the receptor to phagocytose the immune complexes. The phagocytic index was calculated as the number of erythrocytes engulfed per 100 HEK293T cells. (E) Representative microscopy images of HEK293T cells alone or incubated with either opsonized or non-opsonized erythrocytes. External, non-phagocytosed erythrocytes were not removed. Black arrows indicate examples of erythrocytes bound to the membrane of HEK293T cells. Micrographs are representative of three independent experiments. (F) Representative microscopy images of HEK293T cells alone or incubated with either opsonized or non-opsonized erythrocytes upon removal of external, non-phagocytosed erythrocytes by hypotonic (water) lysis. Black arrows indicate examples of erythrocytes inside the cells. The phagocytic index was calculated as the number of erythrocytes engulfed per 100 cells. Micrographs are representative of three independent experiments. In all figures, data are presented as the mean ± standard deviation.
