# Supplementary material for: THP-1 cells transduced with CD16A utilize Fcγ receptor I and III in the phagocytosis of IgG-sensitized human erythrocytes and platelets
Source: PLoS One. 2022 Dec 14;17(12):e0278365. doi: 10.1371/journal.pone.0278365 (PMC9749970; doi:10.1371/journal.pone.0278365)
Supplement: S2 Fig — (DOCX) [file pone.0278365.s002.docx]

**THP-1-CD16A Monocytes**

**A**

**FSC-A**

**SSC-A**

**THP-1-CD16A Macrophages**

**B**

**FSC-A**

**SSC-A**

**Unstained**

**Isotype control**

**THP-1-CD16 Monocytes**

**THP-1-CD16 Macrophages**

**C**

**APC-CD14**

**Count**

**S2 Fig. Flow cytometric analysis of** **size, granularity and CD14 expression on THP-1-CD16A cells.**

Three million cells were cultured in the absence (monocytes) or presence of 100 ng/mL PMA (macrophages). Twenty-four hours later, the media was replaced with fresh complete media, and cells were maintained under the same conditions overnight. Zebra plots showing the size (FSC-A) and granularity (SSC-A) of unstained (**A**) THP-1-CD16A Monocytes and (**B**) THP-1-CD16A Macrophages. (**C**) Histograms representing the expression of CD14 on undifferentiated monocytes or differentiated macrophages that were stained with an APC-conjugated anti-human CD14 antibody. Data are representative of two independent determinations. Flow cytometry was performed using a BD LSRFortessa X-20. Data analysis was performed using FlowJo v10.
