# Supplementary material for: THP-1 cells transduced with CD16A utilize Fcγ receptor I and III in the phagocytosis of IgG-sensitized human erythrocytes and platelets
Source: PLoS One. 2022 Dec 14;17(12):e0278365. doi: 10.1371/journal.pone.0278365 (PMC9749970; doi:10.1371/journal.pone.0278365)
Supplement: S3 Fig — (DOCX) [file pone.0278365.s003.docx]

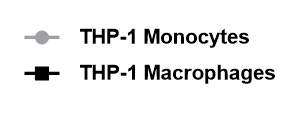

**S3 Fig. Expression of human FcγRs on non-differentiated and PMA-differentiated THP-1 cells.**

THP-1 cells were differentiated to macrophages by treatment with PMA (100 ng/mL). Five hundred thousand cells were stained with direct-labelled monoclonal antibodies against (A) FcγRI (clone 10.1 conjugated to PE/Cy7), (B) FcγRIIA (clone IV.3 conjugated to FITC), (C) FcγRIIA/B/C (clone AT10 conjugated to AF647), or (D) FcγRIIIA (clone 3G8 conjugated to BV421). Stained cells were washed and analyzed by flow cytometry using a BD LSRFortessa X-20. Data analysis was performed using FlowJo v10. Data is presented as the mean ± standard deviation from four independent experiments. MFI: mean fluorescent intensity (arbitrary units). Ab concentration: concentration of the fluorescent antibody for FcγR expression detection. The dashed line represents the MFI value of the corresponding isotype control at 10 µg/mL. The statistical analysis was performed by the non-parametric Mann-Whitney T-test, comparing the MFI of both conditions for each antibody concentration (*: p = 0.05).
