# Supplementary material for: THP-1 cells transduced with CD16A utilize Fcγ receptor I and III in the phagocytosis of IgG-sensitized human erythrocytes and platelets
Source: PLoS One. 2022 Dec 14;17(12):e0278365. doi: 10.1371/journal.pone.0278365 (PMC9749970; doi:10.1371/journal.pone.0278365)
Supplement: S4 Fig — (DOCX) [file pone.0278365.s004.docx]

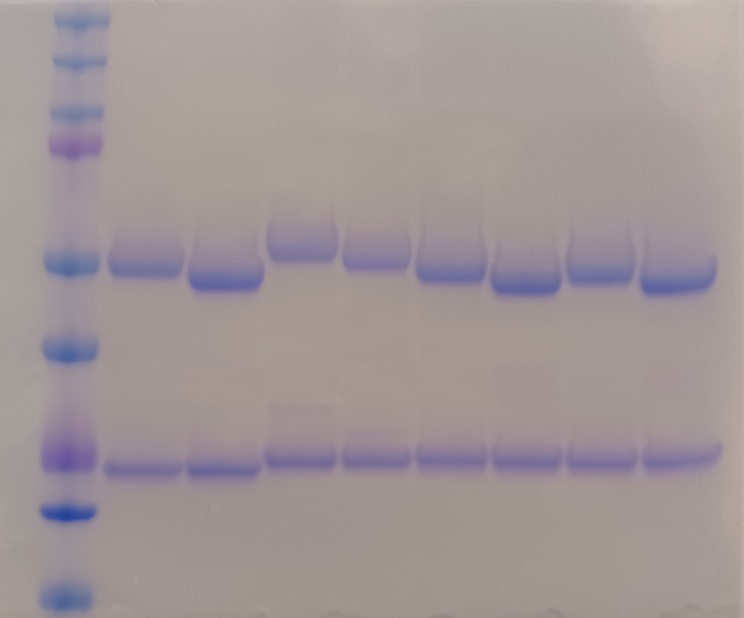


**250 KDa**

**150 KDa**

**100 KDa**

**75 KDa**

**50 KDa**

**37 KDa**

**25 KDa**

**20 KDa**

**10 KDa**

**STDS**

**10.1**

**IV.3**

**AT10**

**3G8**

*****

*****

*****

*****

**S4 Fig.** **SDS-PAGE analysis of blocking antibodies deglycosylation.**

Antibodies 10.1, IV.3, AT10 and 3G8 (0.5 mg/mL) in PBS without calcium and magnesium were incubated with 8 units/μL of glycerol-free recombinant PNGase-F for 48 hours at 37°C. Deglycosylated antibodies were separated from the glycans and the enzyme using a 50 kDa molecular weight cut-off column concentrator with repeated washing with PBS. *: lanes that correspond to deglycosylated antibodies. STDS: molecular weight standards.
